# *PIK3CA* silencing restores anoikis sensitivity in endothelial cells

**DOI:** 10.1007/s11033-026-12680-3

**Published:** 2026-08-29

**Authors:** Diego Mauro Carneiro Pereira, Ana Paula de Sousa Mesquita, Amanda da Silva Cruz, Lucélia Donatti, Helena Bonciani Nader, Carla Cristina Lopes

**Affiliations:** 1 Disciplina de Biologia Molecular, Departamento de Bioquímica, Universidade Federal de São Paulo, Rua Pedro de Toledo, 669, 8º Floor, São Paulo, SP 04039-032 Brazil; 2 Laboratório de Biologia Adaptativa, Departamento de Biologia Celular, Universidade Federal Do Paraná, Curitiba, PR Brazil; 3 Instituto de Ciências Ambientais, Químicas e Farmacêuticas, Universidade Federal de São Paulo, Diadema, SP Brazil

**Keywords:** Cancer, Apoptosis, PI3K, p110α, Anoikis

## Abstract

**Background:**

The development, differentiation, and homeostasis of adherent cells are regulated by interactions among cells, the extracellular matrix (ECM), and soluble factors. Loss of these contacts induces anoikis, a specific form of apoptosis. Tumor cells acquire resistance to anoikis, enabling them to survive without anchorage, invade the vascular system, and colonize distant organs in a process known as metastasis. Activation of the PI3K/Akt signaling pathway is the most common mechanism underlying the acquisition of anoikis resistance in cancer cells. The role of phosphoinositide 3-kinase (PI3K) in cancer is highlighted by the frequent mutation of the gene encoding the p110α catalytic subunit in the most common human cancers. In this study, we aimed to elucidate the role of PI3K (p110α catalytic subunit) in the acquisition of the anoikis-resistant phenotype and in the regulation of cellular characteristics directly related to tumorigenesis.

**Methods and results:**

To this end, we performed *PIK3CA* gene silencing in anoikis-resistant endothelial cells. Consequently, these cells exhibited a reversal of malignancy-associated traits, including morphological characteristics, anoikis resistance, clonogenic potential, proliferation rate, invasiveness, and adhesive capacity, resembling those of wild-type endothelial cells. Furthermore, we observed an increased rate of apoptosis, accompanied by changes in the expression of pro- and anti-apoptotic molecules, as well as increased caspase activation.

**Conclusions:**

In summary, our findings demonstrate that *PIK3CA* silencing reverses key features of the anoikis-resistant endothelial cell phenotype and provides new insights into the molecular mechanisms underlying anoikis resistance.

**Graphical abstract:**

Comparative diagram of the main characteristics of anoikis-resistant endothelial cells before and after *PIK3CA* silencing. Some elements of the graphical abstract were adapted from Servier Medical Art (https://smart.servier.com/, accessed 26 August 2025).

**Figure:**
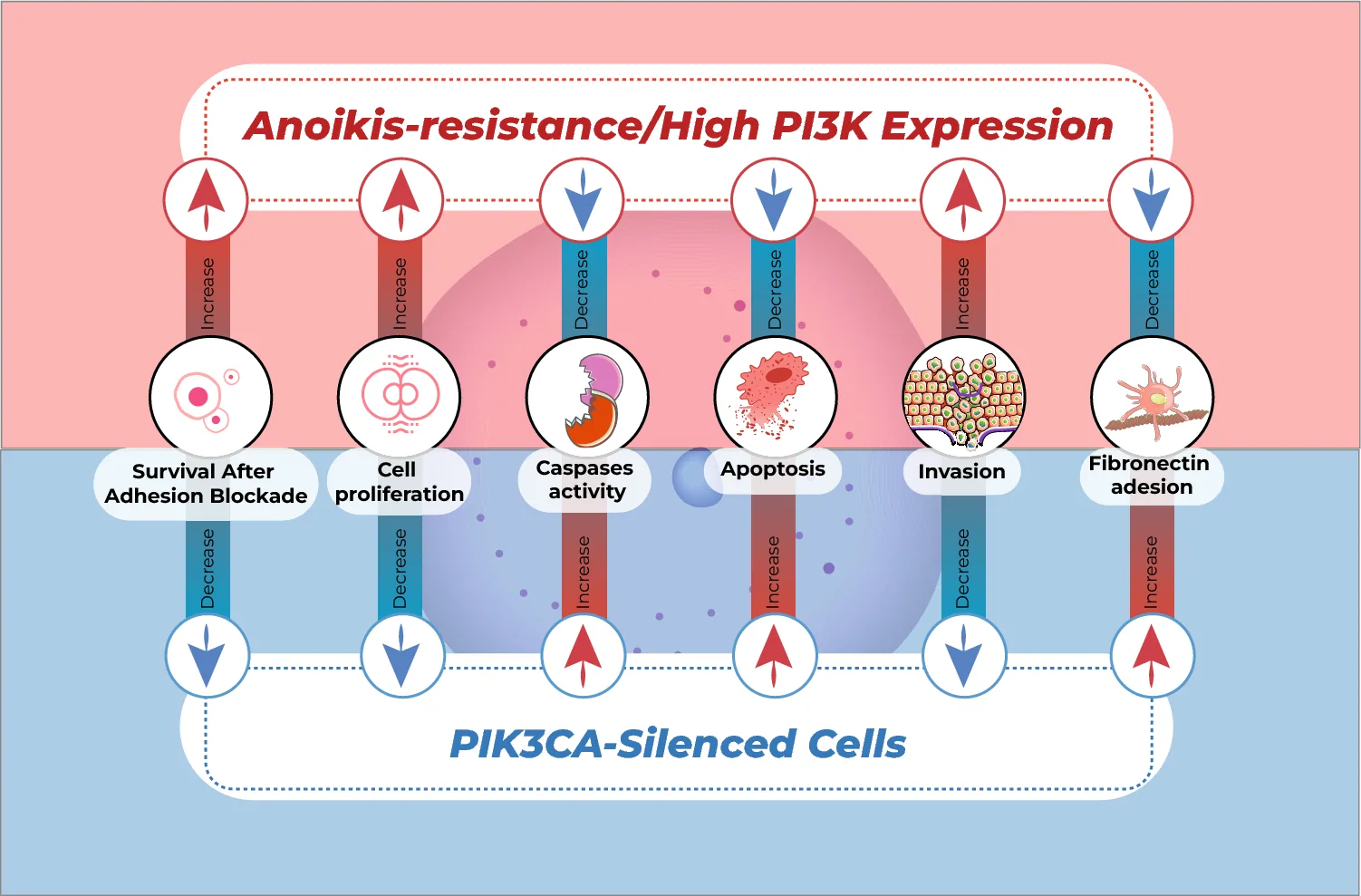

## Introduction

As a barrier to metastatic development, cells normally undergo apoptosis after losing contact with neighboring cells or the extracellular matrix (ECM). This form of cell death is called anoikis [1, 2]. Since its characterization in epithelial and endothelial cells, anoikis has attracted considerable scientific attention, primarily because many tumor cells exhibit resistance to this process, a feature strongly associated with tumor malignancy and aggressiveness [2, 3]. Anoikis resistance is a critical mechanism in tumor metastasis. Tumor cells that acquire this trait can survive after detachment from the primary site, migrate through the vascular system, and eventually colonize distant organs [2, 4, 5].

Anoikis-resistant cells exhibit increased activation of the RAS/ERK and phosphatidylinositol 3-kinase (PI3K)/AKT pathways. These are key signaling cascades involved in regulating cell survival, differentiation, proliferation, and metabolism in response to extracellular cues [6, 7]. PI3K refers to a family of lipid kinases that act as effectors of receptor tyrosine kinases (RTKs) and G protein-coupled receptors (GPCRs). The PI3K/AKT signaling pathway plays a central role in regulating cell survival, proliferation, and cell cycle progression. Its dysregulation is frequently implicated in oncogenesis, tumor progression, and resistance to anticancer therapies [8–10]. The frequent hyperactivation of PI3K in cancer has driven the development of numerous PI3K inhibitors, underscoring its relevance as a promising therapeutic target [11–13].

Most mechanistic studies on anoikis resistance in cancer have been performed using tumor models [3, 14, 15], whereas anoikis-resistant endothelial cell models provide a complementary system for investigating conserved mechanisms of anchorage-independent survival. Despite substantial advances in this field, the specific contribution of individual signaling pathways and their interplay with ECM remodeling remain incompletely understood. This knowledge gap extends to the specific contribution of *PIK3CA*, which encodes the catalytic p110α subunit of PI3K and is one of the most frequently mutated oncogenes in human cancers [16].

Although aberrant PI3K/AKT signaling has been associated with tumor progression, the specific role of *PIK3CA* in regulating anoikis resistance has not been systematically investigated across different experimental models. To better understand the role of *PIK3CA* in the mechanisms underlying the acquisition of anoikis resistance, this study investigated the morphology, behavior, and apoptosis of anoikis-resistant endothelial cells subjected to silencing of the PI3K p110α subunit via transfection with a plasmid vector expressing interfering RNA targeting *PIK3CA* (miR-*PIK3CA*-Adh1-EC). The use of microRNA interference represents a valuable experimental approach for investigating the contribution of specific genes to the malignant phenotype and the molecular mechanisms underlying tumor progression.

## Materials and methods

### Cell lines

The following rabbit aortic endothelial cell lines were used in this study: wild-type endothelial cells (EC), kindly provided by Dr. Buonassisi (University of California, San Diego) and originally established and characterized by Buonassisi and Venter [17], EJ-*ras*-transfected endothelial cells (EJ-*ras* EC), generated in our laboratory from the EC cell line by transfection with the EJ-*ras* oncogene, as previously described by Lopes et al. [18], and anoikis-resistant endothelial cells (Adh1-EC), established in our laboratory from the EC cell line through successive cycles of adhesion blockade, as described by Carneiro et al. [19]. In the present study, three additional cell lines were generated from the Adh1-EC lineage: miR-Empty-Adh1-EC, Adh1-EC cells transfected with a negative control plasmid vector expressing micro interfering RNA; and miR-*PIK3CA*-Adh1-EC 1 and 2, Adh1-EC cells subjected to *PIK3CA* knockdown. The cells were maintained in F12 medium (Sigma-Aldrich, St. Louis, USA) supplemented with 10% fetal calf serum (FCS) (Athena, Campinas, Brazil), penicillin (10,000 U/L), and streptomycin (10 mg/L). Blasticidin (6 µg/mL) was added to the medium of the transfected cell lines. Cells were maintained at 37 °C in an atmosphere of 2.5% CO_2_.

### *PIK3CA* gene silencing using micro RNA interference (miR RNAi)

**Fig. 1:**
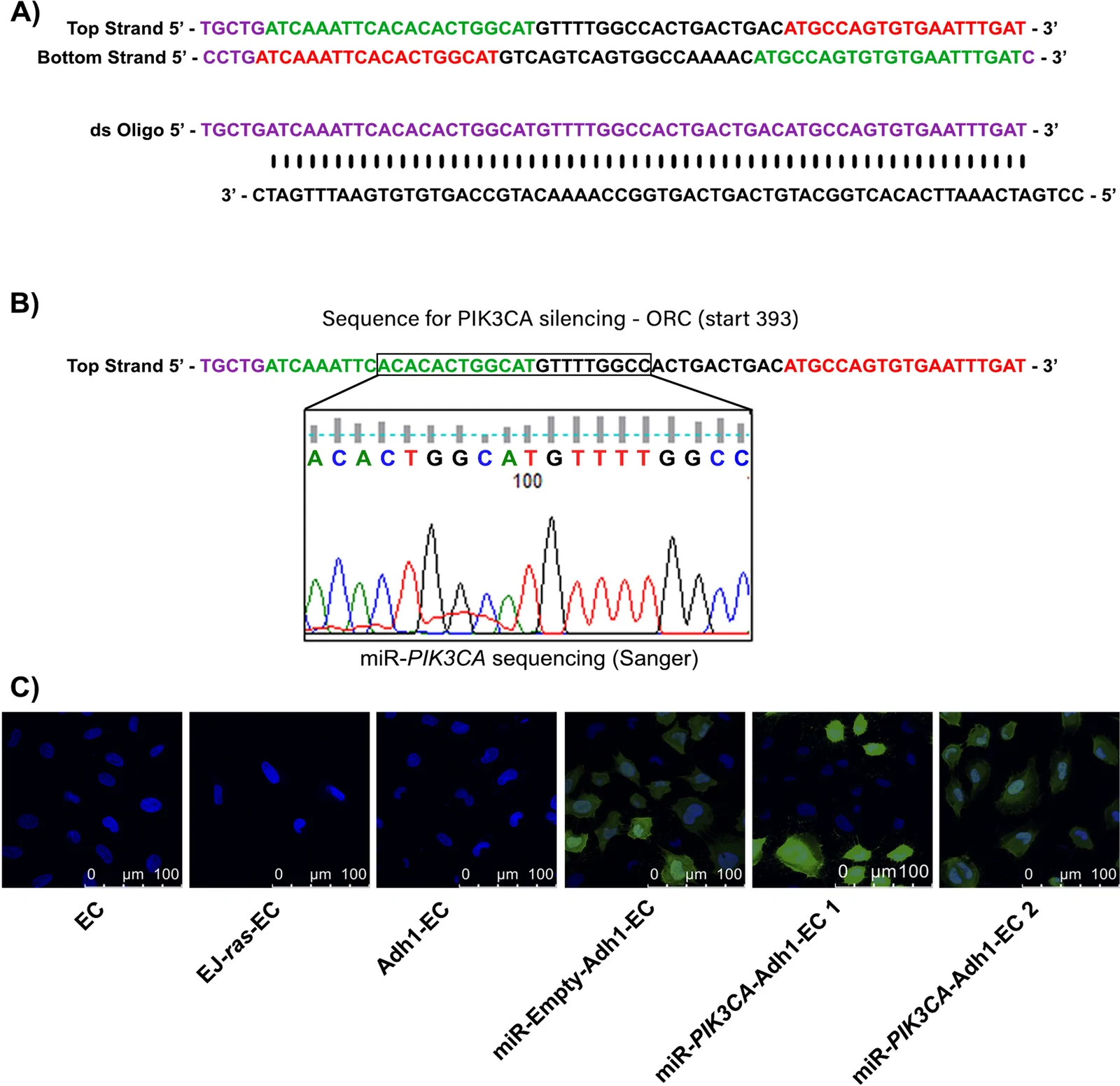
Knockdown of *PIK3CA* gene. (**A**) The pre-miRNA sequences and their predicted secondary structures for targeting *PIK3CA*, including the exact binding sites on the *PIK3CA* mRNA, as well as the double-stranded pre-miRNA oligo cloned into the miRNA expression vector. Green: mature miRNA RNAi sequence. Red: Target sequence. Purple; Linker region. Black: Loop sequence. (**B**) The sequencing was performed using the Sanger technique, and the analysis was carried out using the FinchTV software in order to verify the insert sequence. (**C**) Clones obtained after *PIK3CA* gene silencing. Green fluorescence indicates GFP protein, positive control for plasmid transfection, nuclei were stained with DAPI (blue). Images obtained with 40× objective. Adh1‐EC, anoikis‐resistant endothelial cells; EC, rabbit aorta endothelial cells; EJ‐*ras* EC, EJ‐*ras* transfected endothelial cells; miR‐Empty‐Adh1‐EC, Adh1‐EC cells transfected with Neg‐miRNA; miR‐*PIK3CA*‐Adh1‐EC, Adh1‐EC cells transfected with miRNA‐*PIK3CA*

The Adh1-EC cell line stably expressing *PIK3CA* microRNA (miR-*PIK3CA*-Adh1-EC) was generated using the BLOCK-iT™ Pol II miR RNAi Expression Vector Kit (Invitrogen, Catalog No. K493600) according to the manufacturer’s instructions. Synthesized oligonucleotides (IDT, Coralville, USA) targeting the *Oryctolagus cuniculus PIK3CA* sequence (XM_008266536.1), 5′- TGCTGATCAAATTCACACACTGGCATGTTTTGGCCACTGACTGACATGCCAGTGTGAATTTGAT-3′ (top strand) and 5′- CCTGATCAAATTCACACTGGCATGTCAGTCAGTGGCCAAAACATGCCAGTGTGTGAATTTGATC-3′ (bottom strand), were cloned into the pcDNA 6.2-GW/EmGFP-miR expression vector. This vector expresses micro interfering RNA (miRNA) in mammalian cells under the control of the human cytomegalovirus immediate-early promoter. Gene sequencing using the Sanger method was performed to confirm sequence insertion (Fig. 1b) [20]. The vector also expresses emerald green fluorescent protein (EmGFP) as a reporter gene, and the miRNA-coding sequence insertion site is located in the 3′ untranslated region of the EmGFP mRNA (Fig. 1c).

### Cell viability assay

All cell lines were harvested using pancreatin, and 5 × 10^4^ cells were cultured in 5 mL of F12 medium on 1% (w/v) agarose-coated plates for 96 h. After 96 h, the supernatant was collected and centrifuged at 1,500 rpm for 5 min. The pellet was resuspended in 500 μL of F12 medium supplemented with 10% FBS and distributed into five wells of a 96-well plate with transparent walls, followed by incubation at 37 °C for 24 h. To evaluate the viability of cells that remained viable after adhesion blockade, the culture medium was removed and replaced with 100 μL of F12 medium containing resazurin (alamarBlue®) at 0.05 mg/mL. The cells were incubated at 37 °C in 2.5% CO₂ for 4 h, and absorbance was measured by spectrophotometry at 570 and 600 nm.

### RNA extraction and real-time reverse transcription–PCR analysis

Total RNA was isolated using Ribozol reagent (Amresco LLC, OH) according to the manufacturer’s instructions. Subsequently, cDNA was synthesized using the All-in-One cDNA Synthesis SuperMix (Bimake, USA), and gene expression was quantified using the Maxima SYBR Green qPCR Master Mix Kit (Thermo Fisher Scientific, USA). Reactions were carried out on an ABI 7500 Real-Time PCR System (Applied Biosystems, USA) according to the manufacturer’s instructions. The primers employed in this study were: *PIK3CA* Fwd (5′-AGC AAG CGC CCC AAA CTG GAA3′) and *PIK3CA* Rev (5′-TGC AGG CCA CTG GTG AAG CAA-3′); *BCL2* Fwd (5′-AGC TCA CTG TGT CTC TGT GC-3′) and *BCL2* Rev (5′-GGG CTA GGT CTC CAG GTG AT-3′); *BAX* Fwd (5′- GCC TCA CTC ACC ATC TGG AAG-3′) and *BAX* Rev (5′-AGA AAA ACG CCT GTG TCC C-3′); *BAD* Fwd (5′-CCG AAG GAT GAG CGA CGA G-3′) and *BAD *Rev (5′-CCC ACC AAG ACT GGA TGA CG-3′); *Caspase 3* Fwd (5′-GAG ACA GAC AGT GGG GTT GAC-3′) and *Caspase 3 *Rev (5′-CGT ACT CTT TCA GCA TGG CAC-3′); *GAPDH* Fwd (5′-CGC TTC GCT CTC TGC TCC TCC-3′) and *GAPDH* Rev (5′-TGG TGA CCA GGC GCC CAA TAC-3′). qPCR amplification was carried out for 40 cycles under the following conditions: 95 °C for 15 s and 60 °C for 60 s. Relative mRNA expression levels were calculated using the comparative threshold cycle (Ct) method (2^−ΔΔCt), with *GAPDH* as the endogenous control for normalization of mRNA expression levels, thereby accounting for variations in sample loading. Melting curve analysis was performed after amplification to verify the specificity of the PCR products and exclude nonspecific amplification or primer-dimer formation. All qPCR reactions were conducted in technical triplicate and independently repeated three times.

### Western blotting

Cell lysis was carried out for 2 h at 4 °C in buffer containing 50 mM Tris–HCl (pH 7.4), 1% Tween 20, and Halt Protease and Phosphatase Inhibitor Cocktail (Thermo Fisher Scientific, USA). The lysates were centrifuged at 18,000 × *g* for 5 min, and the supernatants were collected. Protein concentrations were determined using the BCA Protein Assay Kit (Thermo Fisher Scientific, USA). Protein extracts (20 µg per sample) were resolved by 10% SDS–PAGE and transferred to PVDF membranes (Merck Millipore, USA). Following membrane blocking, the membranes were incubated overnight at 4 °C with the following primary antibodies diluted in blocking buffer: rabbit anti-PI3K p110α (Millipore, Cat. #04–399, 1:1000), rabbit anti-BAD (Millipore, Cat. #04–432, 1:500), rabbit anti-caspase−3 (Abcam, Cat. #ab44976, 1:500), rabbit anti-Bcl-2 (Cell Signaling, Cat. #2870, 1:1000), and mouse anti-β-actin (Cell Signaling, Cat. #3700, 1:1000). Membranes were then incubated with secondary antibodies (1:10,000) for 1 h at room temperature. Protein bands were visualized by enhanced chemiluminescence using the SuperSignal West Pico Chemiluminescent Substrate (Thermo Fisher Scientific, USA). Signal acquisition and densitometric analysis were performed using the Alliance Mini imaging system (UVITEC, Cambridge, UK) and UVIBAND MAX v1503b software (UVITEC, Cambridge, UK), respectively. All experiments were performed in duplicate and independently repeated three times.

### Immunocytochemistry

For immunofluorescence analysis, endothelial cells were seeded onto 13 mm glass coverslips at a density of 5 × 10^3^ cells per coverslip in 24-well plates and maintained for 48 h. Cells were then fixed with 4% formaldehyde in PBS (pH 7.4) for 20 min at room temperature, followed by four washes with PBS and one wash with PBS supplemented with 0.1 M glycine. The cells were then permeabilized with PBS containing 1% BSA and 0.01% saponin for 15 min at room temperature. After washing, the cells were incubated with an anti-PI3K antibody (Millipore, Cat. #04–399, 1:200) diluted in PBS containing 0.01% saponin for 1 h at room temperature. Subsequently, the samples were washed and incubated with an Alexa Fluor 594-conjugated goat anti-rabbit secondary antibody (1:250; Molecular Probes) for 2 h. F-actin was visualized using Alexa Fluor 488 Phalloidin and Alexa Fluor 594 Phalloidin (1:200; Molecular Probes). Nuclear staining was performed with DAPI (4′,6-diamidino-2-phenylindole dihydrochloride; 1:10,000). Following the final washes, coverslips were mounted in Fluoromount-G diluted in PBS (2:1) and analyzed using an inverted confocal laser-scanning microscope (Leica TCS SP8, Germany). Representative images presented in the Results section were selected from two independent experiments.

### Scanning electron microscopy

For scanning electron microscopy, 5 × 10^3^ endothelial cells were seeded onto coverslips as described in the Immunocytochemistry section. After the incubation period, the cells were washed with PBS and fixed for 30 min in Karnovsky’s fixative (2% paraformaldehyde and 2.5% glutaraldehyde in 0.2 M cacodylate buffer, pH 7.2). Following washes with 0.1 M cacodylate buffer (pH 7.2), the samples were dehydrated through a graded ethanol series. The samples were subjected to critical-point drying using a Bal-Tec CPD-030, followed by gold sputter coating with a Balzers SCD-030. Ultrastructural analysis was then performed at the Electron Microscopy Center of the Federal University of Paraná (UFPR) using a TESCAN VEGA 3 LMU scanning electron microscope (Tescan Group, Brno-Kohoutovice, Czech Republic).

### Flow cytometry

For flow cytometry analysis, cells were fixed in 2% paraformaldehyde for 30 min, centrifuged at 2,000 rpm for 5 min, and washed with 0.1 M glycine. Permeabilization was then carried out with PBS containing 1% BSA and 0.1% saponin for 10 min. The cells were subsequently incubated with the primary antibody for 1 h at 4 °C, followed by incubation with the secondary antibody. Fluorescence was measured using a FACSCalibur flow cytometer (Becton Dickinson, CA, USA). Data acquisition was performed using CellQuest software (BD Biosciences, USA), and fluorescence data were analyzed using FlowJo version 10.0 (FlowJo LLC, USA).

### Cell growth analysis

A total of 5 × 104 cells were cultured in F12 medium (Thermo Fisher Scientific) supplemented with 10% fetal bovine serum (FBS; Athena, Brazil) at 37 °C in 2.5% CO₂. Every 48 h, the cells were fixed with absolute ethanol, stained with DAPI (1:5,000) diluted in PBS, and stored at 4 °C. On day 8, images of the wells were acquired and quantified using the IN Cell Analyzer 2200 imaging system (GE Healthcare Life Sciences). Triplicate wells were analyzed at each time point (0, 2, 4, 6, and 8 days), and all experiments were independently repeated three times.

### Annexin V-Cy5/PI staining assay

Apoptosis was evaluated by annexin V-Cy5 binding to phosphatidylserine exposed on the outer leaflet of the plasma membrane during early apoptosis. Cells and their derived clones (1 × 10^6^ cells) were resuspended in the binding buffer supplied with the Annexin V-Cy5 Apoptosis Detection Kit (Abcam, USA) and incubated with 5 µL of annexin V-Cy5 and 5 µL of propidium iodide (PI) for 30 min at room temperature in the dark. Stained cells were analyzed by flow cytometry using a FACSCalibur (Becton Dickinson and Co., Franklin Lakes, NJ, USA), and the data were processed using FlowJo software (Tree Star, USA). All experiments were independently repeated three times.

### Adhesion assays

For adhesion assays, 96-well culture plates (Life Sciences, USA) were coated with fibronectin (FN), collagen IV (Col IV), or laminin (LN) (10 µg/mL) for 2 h at 37 °C. The wells were washed with PBS and blocked with 1% BSA in PBS for 1 h at 37 °C. Cells (5 × 10^3^ cells/well) suspended in F12 medium were seeded onto the coated plates and allowed to adhere for 2 h at 37 °C. Non-adherent cells were removed by gentle washing with PBS, and the attached cells were fixed with methanol for 20 min. After fixation, the methanol was aspirated, the wells were rinsed with PBS, and the nuclei were stained with DAPI (1:5,000) diluted in PBS. Images were acquired and quantified using the IN Cell Analyzer 2200 system (GE Healthcare Life Sciences). All experiments were performed in triplicate and independently repeated three times on separate days.

### Invasion assay

Transwell inserts (24-well) with 8 µm pore-size polycarbonate membranes (Life Sciences, USA) were coated with ECL cell attachment matrix (1 mg/mL; entactin, LN, and Col IV; Merck Millipore, USA). Prior to seeding, the coated inserts were equilibrated with prewarmed serum-free F12 medium for 2 h at 37 °C in 2.5% CO₂. Cells (5 × 10^4^) suspended in serum-free F12 medium were added to the upper chamber, while the lower chamber was filled with F12 medium supplemented with 10% FCS as a chemoattractant. After incubation for 48 h at 37 °C, non-invading cells were gently removed from the upper surface of the membrane using a cotton swab. Cells that had migrated through the membrane were fixed with 1% formaldehyde for 30 min at room temperature and stained with 1% toluidine blue (Sigma-Aldrich, St. Louis, MO, USA) prepared in 1% sodium tetraborate for 15 min. Invaded cells were visualized under a light microscope (Carl Zeiss, Jena, Germany), and stained cells were quantified in 10 random fields at 20× magnification. All experiments were independently repeated three times.

### Colony formation assay

To investigate the ability of the silenced cells to survive and form colonies after adhesion blockade, approximately 5 × 10^4^ endothelial cells were plated on 100 mm plates coated with a thin layer of 1% agarose. The cells were maintained in suspension at 37 °C in an atmosphere of 2.5% CO₂ for 96 h. After 96 h, the suspended cells were cultured under adherent conditions in F12 medium supplemented with 10% FBS in 60 mm plates to allow adhesion and colony formation. On day 5, the colonies were rinsed with PBS, fixed with 4% paraformaldehyde for 15 min, and stained with 1% toluidine blue for 5 min. Excess dye was removed by washing with PBS. To quantify viable cells, the bound dye was solubilized by cell lysis in 1% SDS, and absorbance was measured at 570 nm using a microplate reader.

### Caspase 3/7 and 8 activity

Cells were seeded into white-walled 96-well plates at a density of 5 × 104 cells per well in triplicate. Caspase-Glo 3/7 or Caspase-Glo 8 reagent (Promega) was then added, and the samples were incubated according to the manufacturer’s protocol. Luminescence was measured using a VICTOR™2 multilabel reader.

### Statistical analysis

Analysis of data was performed using the GraphPad Prism 6 software. All data are expressed as the mean ± standard error of the mean. Statistical significance was assessed using one-way ANOVA. *P* values < 0.05 were considered statistically significant.

## Results

### Gene silencing of *PIK3CA* via miR RNAi effectively reduces its expression in endothelial cells

Hyperactivation of the PI3K/mTOR/AKT signaling pathway is one of the most common pathological alterations in human cancers [21]. In this study, we aimed to elucidate the role of the p110α catalytic subunit of PI3K in the acquisition of the anoikis-resistant phenotype and in the regulation of cellular features directly associated with tumorigenesis. Accordingly, gene silencing of *PIK3CA*, the gene encoding this protein, was performed.

After achieving stable knockdown of *PIK3CA* and confirming sequence insertion by gene sequencing, expression of the reporter gene EmGFP was verified by immunofluorescence and confocal microscopy (Fig. 1).

In a previous study by our group, we demonstrated that anoikis-resistant endothelial cells exhibited increased gene and protein expression of the PI3K/AKT pathway compared with the control group [6]. To investigate the role of the PI3K pathway in promoting anoikis resistance, we developed a microRNA-based interference strategy to selectively downregulate *PIK3CA* expression in anoikis-resistant endothelial cells.

**Fig. 2:**
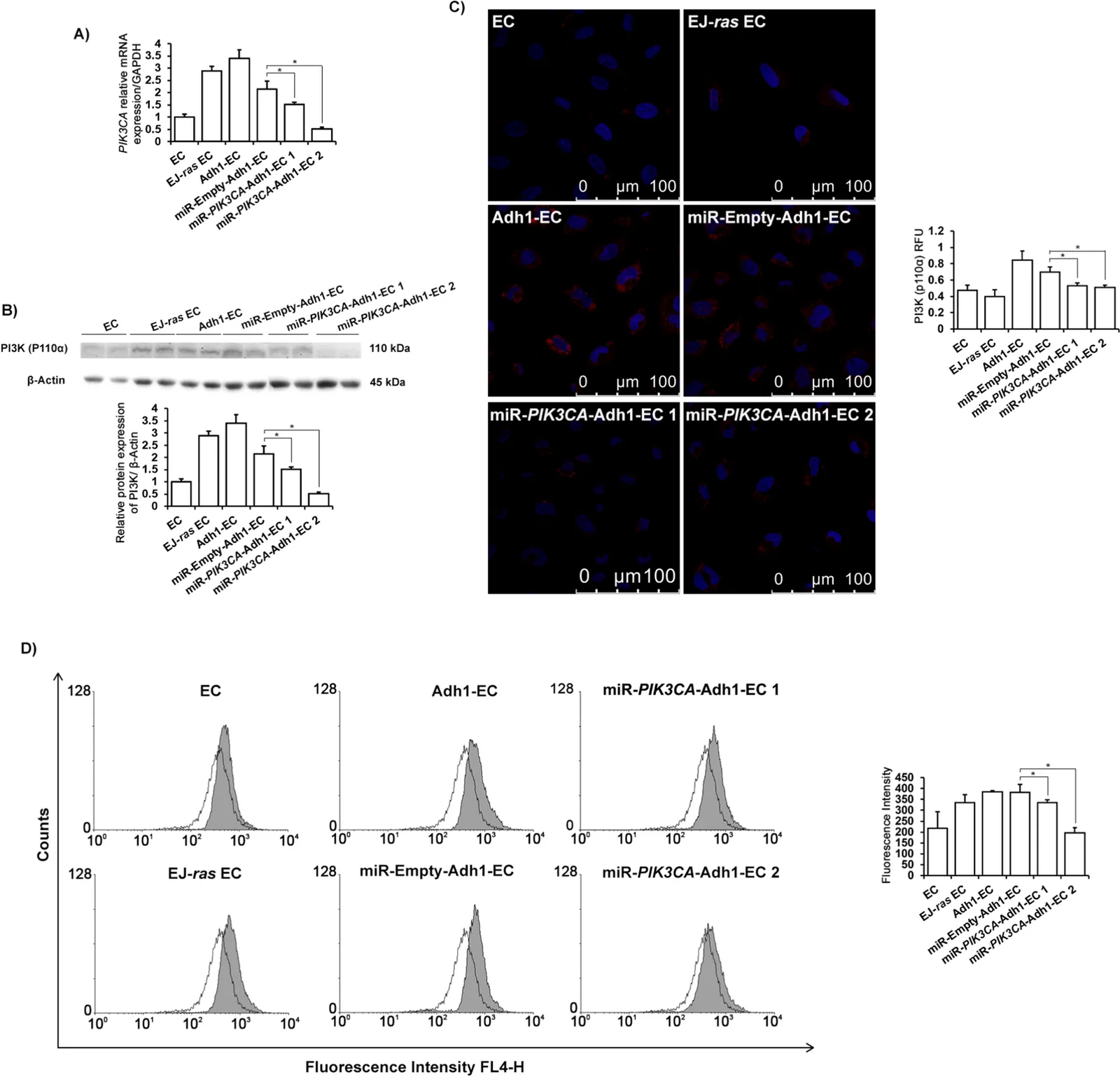
Gene and protein expression of PI3K (p110α) in EC-derived cell lines. (**A**) *PIK3CA* mRNA expression levels. (**B**) Protein expression of PI3K by western blot. β-Actin was used as a loading control. (**C**) Immunofluorescent analysis of PI3K (red). Nucleus was stained with DAPI (blue). (**D**) Flow cytometry analysis illustrating PI3K expression. All experiments were independently repeated three times. Each independent experiment was performed in technical triplicate, except for western blot analysis, which was performed in duplicate. Adh1‐EC, anoikis‐resistant endothelial cells; EC, rabbit aorta endothelial cells; EJ‐*ras* EC, EJ‐*ras* transfected endothelial cells; miR‐Empty‐Adh1‐EC, Adh1‐EC cells transfected with Neg‐miRNA; miR‐*PIK3CA*‐Adh1‐EC, Adh1‐EC cells transfected with miRNA‐*PIK3CA*. All experiments were performed in triplicate. Data are reported as means ± SE. *p ≤ 0.05

The efficiency of *PIK3CA* silencing was validated by western blotting, real-time PCR, and immunofluorescence, following the protocols described in the Materials and Methods section. *PIK3CA* mRNA and PI3K (p110α) protein levels were assessed across several cell lines: wild-type cells (EC), EJ-*ras*-transfected endothelial cells (EJ-*ras* EC), the anoikis-resistant cell line (Adh1-EC), a negative control anoikis-resistant cell line transfected with a non-targeting miRNA (miR-Empty-Adh1-EC), and two *PIK3CA*-silenced cell lines (miR-*PIK3CA*-Adh1-EC 1 and miR-*PIK3CA*-Adh1-EC 2). As illustrated in Fig. 2, *PIK3CA* mRNA expression in the silenced Adh1-EC cells was reduced compared with the parental Adh1-EC cells. Furthermore, the two silenced cell lines exhibited decreases in PI3K protein expression of 56% and 83%, respectively (Fig. 2). The knockdown was specific, as no significant differences in β-actin or *GAPDH* expression were observed between the silenced cells and the controls.

### Silencing of *PIK3CA* gene induces morphological changes in anoikis-resistant endothelial cell

To evaluate morphological changes, scanning electron microscopy was performed. The data showed that the EJ-*ras* EC and Adh1-EC cell lines exhibited a higher number of non-adherent cells, whereas the miR-*PIK3CA*-Adh1-EC cell line resembled the wild-type EC cells, with cells distributed across the coverslip. Adhesion assays were subsequently performed to further investigate these observations. In addition, wild-type EC cells exhibited normal cell spreading, with prominent protrusive structures, such as filopodia and lamellipodia, on the cell surface. In contrast, Adh1-EC and EJ-*ras* EC cells showed reduced formation of these protrusions, as well as decreased cell spreading. These characteristics were restored in the *PIK3CA*-silenced cells, indicating morphological restoration.

**Fig. 3:**
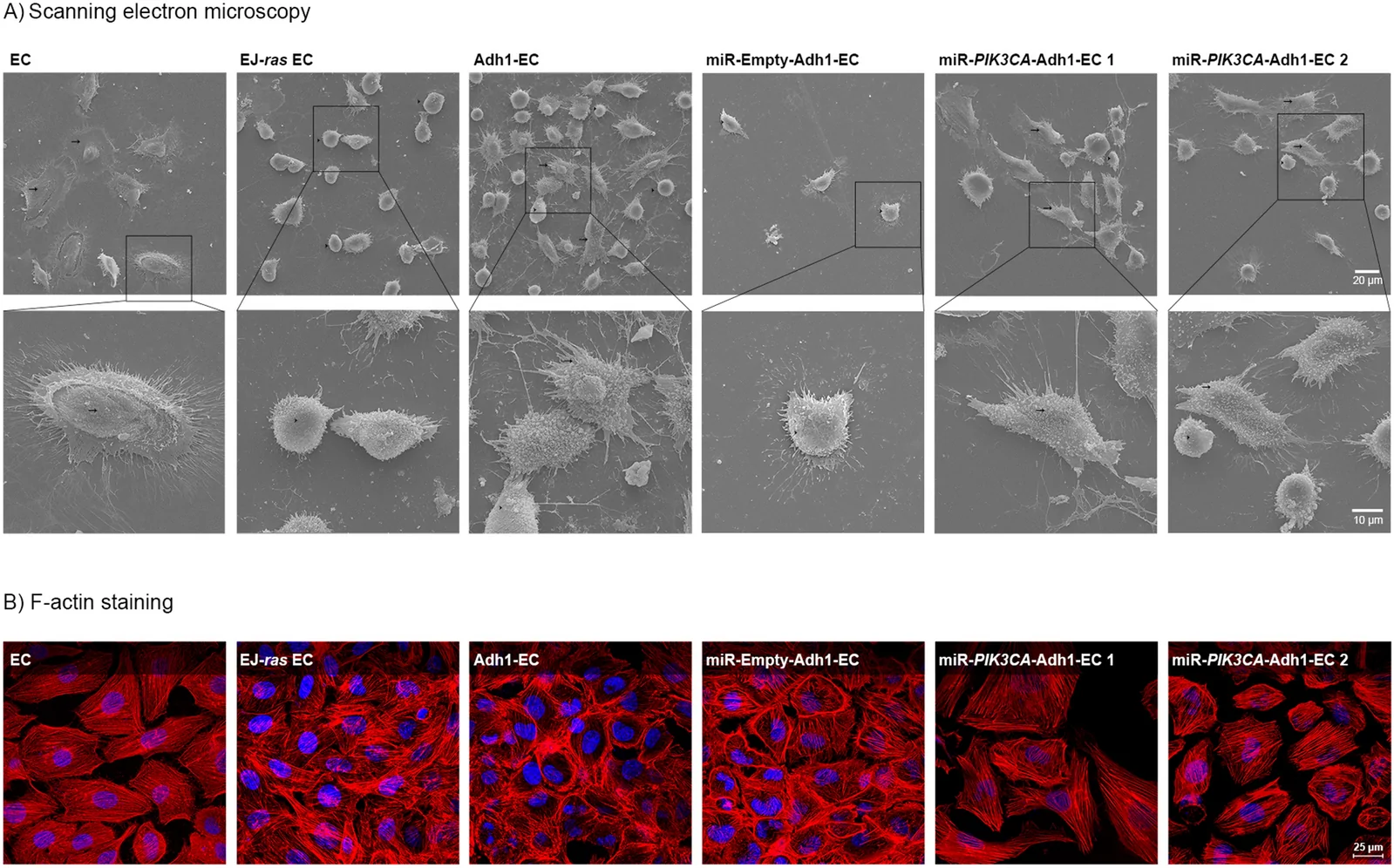
Analysis of morphology of anoikis-resistant endothelial cells after *PIK3CA* gene silencing. (**A**) Scanning electron microscopy of different lineages derived of rabbit aortic endothelial cells. Panels 1 and 2: 1500× and 5000× magnification, respectively. The arrows indicate the cells with the most protrusions and scattering and the arrowhead indicates the less scattered cells. (**B**) Expression and localization of F-actin in anoikis-resistant endothelial cells after *PIK3CA* gene silencing. F-actin was labeled with Alexa Fluor® 647-conjugated phalloidin Media Play Pause (red), and nuclei were stained with DAPI (blue). All experiments were independently repeated two times. Images obtained with 63× objective. Scale bars are located in the lower right corner of the figure. Adh1‐EC, anoikis‐resistant endothelial cells; EC, rabbit aorta endothelial cells; EJ‐*ras* EC, EJ‐*ras* transfected endothelial cells; miR‐Empty‐Adh1‐EC, Adh1‐EC cells transfected with Neg‐miRNA; miR‐*PIK3CA*‐Adh1‐EC, Adh1‐EC cells transfected with miRNA‐*PIK3CA*

Immunofluorescence was performed to assess the distribution of actin filaments in the cell lines. The phalloidin used for staining binds specifically to filamentous actin (F-actin), but not to actin monomers. Fig. 3 shows the distribution of F-actin in the analyzed cells. The red-stained structures represent F-actin, whereas the blue-stained regions indicate the cell nuclei. In contrast to the parental Adh1-EC cells, the *PIK3CA*-silenced cells exhibited a more regular morphology and reorganization of actin filaments, with more widely distributed actin fibers.

### Downregulation of *PIK3CA* suppresses invasive behavior and clonogenic potential of anoikis-resistant endothelial cells

**Fig. 4:**
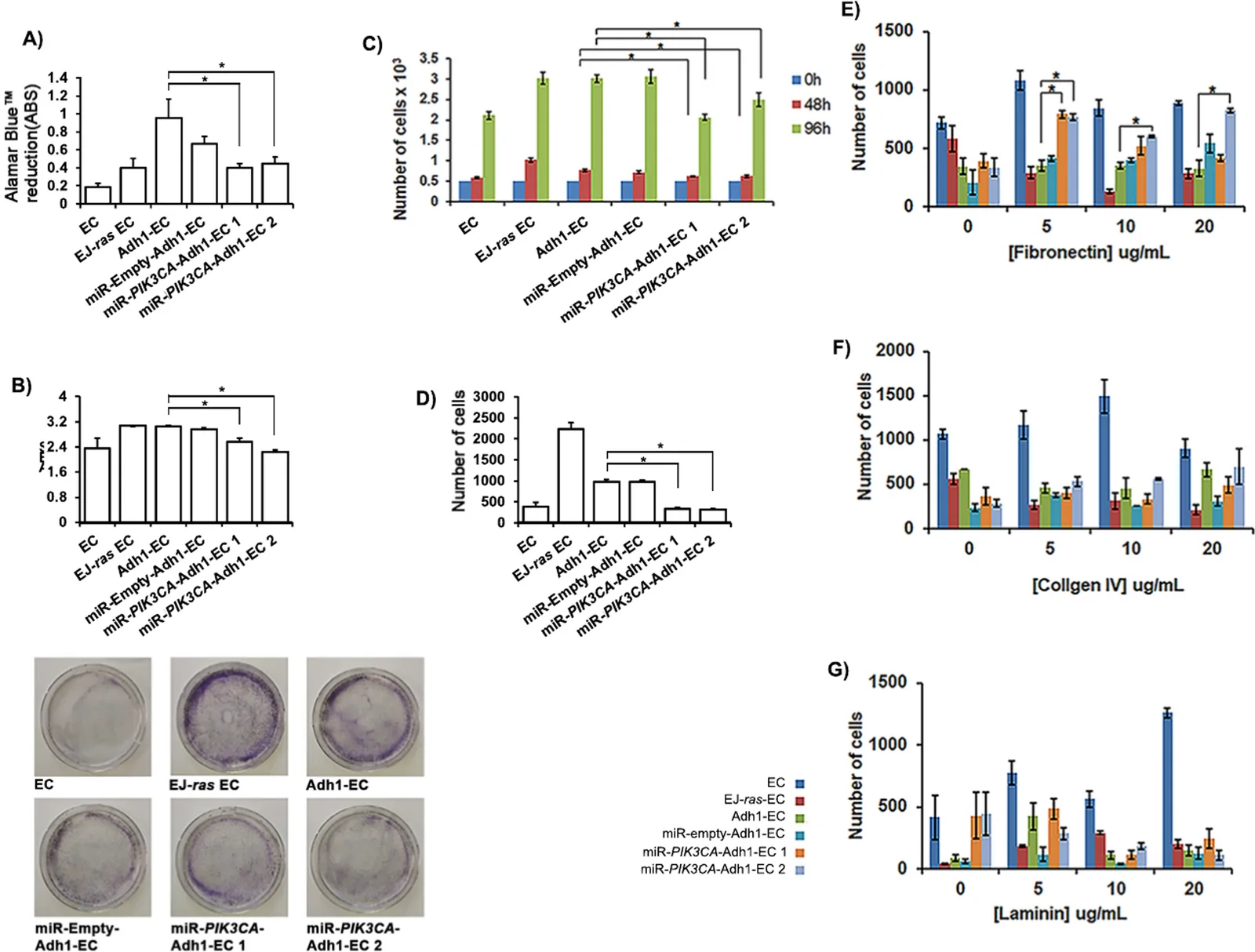
Behaviors of anoikis-resistant cells after *PIK3CA* gene silencing. (**A**) Cell viability following adhesion blockade was evaluated using the redox-based resazurin assay (alamarBlue®). (**B**) Colony formation was assessed five days after adhesion blockade. (**C**) Cell proliferation was evaluated under standard culture conditions. (**D**) Invasion activity of EC-derived cell lines was analyzed by using a Transwell coated with ECL cell attachment matrix. (**E–G**). Cell adhesion to extracellular matrix components was quantified using fibronectin (**E**), collagen IV (**F**), and laminin (**G**) coatings, respectively. Adh1‐EC, anoikis‐resistant endothelial cells; EC, rabbit aorta endothelial cells; EJ‐*ras* EC, EJ‐*ras* transfected endothelial cells; miR‐Empty‐Adh1‐EC, Adh1‐EC cells transfected with Neg‐miRNA; miR‐*PIK3CA*‐Adh1‐EC, Adh1‐EC cells transfected with miRNA‐*PIK3CA*. All experiments were performed in triplicate, independently repeated three times. Data are reported as means ± SE. *p ≤ 0.05

Cell viability following adhesion blockade was assessed by the redox activity of resazurin (alamarBlue®) (Fig. 4A). Silencing of *PIK3CA* led to a significant reduction in cell viability after adhesion blockade, suggesting that the silenced cell lines became more susceptible to anoikis.

Furthermore, the colony-forming capacity after adhesion blockade was evaluated. As shown in Fig. 4B, Adh1-EC and EJ-*ras* EC cell lines were able to evade apoptotic mechanisms, surviving in the absence of adhesion while maintaining their proliferative capacity. This behavior reflects a malignant phenotype characterized by an increased ability to form colonies compared with wild-type EC cells. Notably, *PIK3CA* silencing in anoikis-resistant cells significantly impaired this ability, reducing the colony formation rate (Fig. 4B). Regarding cell proliferation, the results presented in Fig. 4C show that anoikis-resistant endothelial cells (Adh1-EC) exhibit a higher proliferation rate than wild-type EC cells. *PIK3CA* silencing significantly reduced the proliferative capacity of these cells, with the most pronounced effect observed at the 96 h time point.

To assess the invasive potential of the cell lines, an invasion assay was performed using a matrix protein mixture containing entactin, Col IV, and LN. Anoikis-resistant endothelial cells exhibited high invasive capacity, comparable to that of endothelial cells transfected with the EJ-*ras* oncogene (EJ-*ras* EC) Fig. 4D. The results show that *PIK3CA* silencing significantly reduced the invasive capacity of miR-*PIK3CA*-Adh1-EC cells compared with the parental Adh1-EC cell line.

Adhesion assays were performed using different concentrations of FN, Col IV, and LN as substrates for all cell lines (Fig. 4E-G). *PIK3CA* silencing increased adhesion to FN at concentrations of 0, 5, 10, and 20 µg/mL. No significant changes were observed in adhesion to Col IV or LN in the silenced cells compared with the anoikis-resistant cells.

### *PIK3CA* knockdown enhances caspase activation and apoptosis rate in anoikis-resistant endothelial cells

**Fig. 5:**
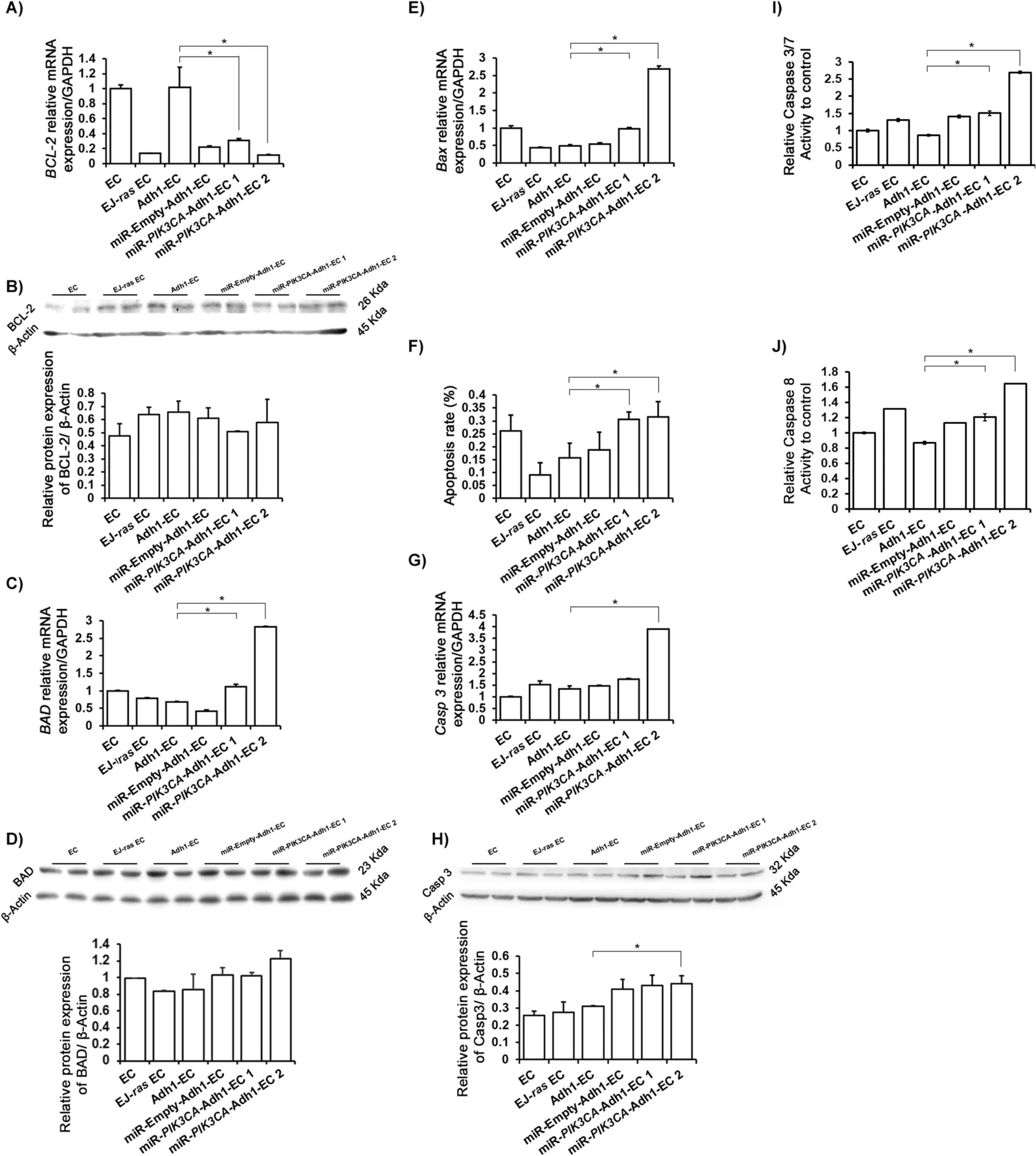
Evaluation of apoptosis-related markers in EC-derived cell lines. (**A**) mRNA expression of *BCL-2*; (**B**) Protein levels of BCL-2; (**C**) mRNA expression of *BAD*; (**D**) Protein levels of BAD; (**E**) mRNA expression of *Bax*; (**F**) Apoptosis rate in EC-derived cell lines; (**G**) mRNA expression of *Casp 3*; (**H**) Protein levels of Casp 3; (**I**) Casp-3/7 activity; (**J**) Casp-8 activity. Casp = Caspase. Adh1‐EC, anoikis‐resistant endothelial cells; EC, rabbit aorta endothelial cells; EJ‐*ras* EC, EJ‐*ras* transfected endothelial cells; miR‐Empty‐Adh1‐EC, Adh1‐EC cells transfected with Neg‐miRNA; miR‐*PIK3CA*‐Adh1‐EC, Adh1‐EC cells transfected with miRNA‐*PIK3CA*. All experiments were independently repeated three times. Each independent experiment was performed in technical triplicate, except for western blot analyses, which were performed in duplicate. Data are reported as means ± SE. *p ≤ 0.05

Analysis of *BCL2* mRNA expression revealed a reversal of the phenotype in *PIK3CA*-silenced cells, leading to a decrease in *BCL2* mRNA levels and a concomitant upregulation of *BAD *expression (Fig. 5A and C). At the protein level, no significant changes were observed in BCL2 or BAD expression (Fig. 5B and D). Regarding *BAX*, an increase in mRNA expression was observed in the silenced cells (Fig. 5E). Collectively, these data suggest that the balance between pro- and anti-apoptotic factors, particularly the regulation of BCL2, BAD, and *BAX*, is functionally associated with the induction of apoptosis. To confirm this, the apoptotic rate was further assessed.

Consistent with our hypothesis, anoikis-resistant cells subjected to *PIK3CA* silencing exhibited higher apoptotic rates than the parental Adh1-EC cells (Fig. 5F). As promoters of apoptosis, caspases (cysteine-dependent aspartate-specific proteases) cleave their substrates at aspartic acid residues. As a protective mechanism against inadvertent activation, these enzymes are synthesized as inactive precursors (zymogens) that require proteolytic cleavage for activation [22]. Assessing caspase−8 activity is particularly relevant, as it serves as an early marker of apoptosis initiation through the extrinsic pathway, which is triggered by death receptor activation. Similarly, evaluation of caspase−3/7 activity is important not only as an indicator of intrinsic pathway activation but also as a mechanism for amplifying signals originating from the extrinsic pathway.

Subsequently, *CASP3* mRNA and caspase−3 protein levels (Fig. 5G-H), as well as caspase−3/7 and caspase−8 activities, were assessed to confirm the involvement of caspases in mediating the apoptotic process (Fig. 5I-J). The silenced cells displayed increased *CASP3* mRNA and caspase−3 protein levels, as well as increased caspase−3/7 and caspase−8 activities, compared with Adh1-EC cells. Together, these findings support the involvement of both the extrinsic and intrinsic apoptotic pathways following *PIK3CA* silencing.

## Discussion

The present study demonstrates that *PIK3CA* plays a central role in maintaining the malignant phenotype of anoikis-resistant endothelial cells. Silencing *PIK3CA* promoted the reversal of several malignant characteristics, including restoration of a more spread cell morphology, reduced cell viability following adhesion blockade, increased adhesion to FN, and enhanced apoptotic activity. Resistance to anoikis has been associated with defects in apoptotic signaling, particularly in caspase activation [23]. Consistent with this mechanism, *PIK3CA*-silenced anoikis-resistant endothelial cells exhibited increased caspase−3 expression, enhanced caspase−3/7 and − 8 activities, and a higher apoptotic rate, indicating recovery of apoptotic capacity.

The morphological changes observed after *PIK3CA* silencing may indicate a reversion of cellular architecture, restoring the original endothelial cell characteristics, in which proper adhesion and organization are essential for tissue function. These findings suggest that the PI3K pathway plays a role in cytoskeletal reorganization in endothelial cells. During cell movement associated with metastasis, cytoskeletal remodeling is required. The regulation of the cytoskeleton during migration involves both biochemical factors and mechanotransduction signals derived from the ECM [24]. Activation of the PI3K signaling pathway plays a crucial role in the reorganization of actin filaments, directly influencing the motility and migratory potential of cancer cells [25, 26].

These morphological changes were accompanied by functional alterations. *PIK3CA* silencing promoted anoikis, as evidenced by reduced cell viability following adhesion blockade. This is consistent with previous reports showing that the PI3K/AKT signaling pathway, through IGF1R, promotes cellular transformation and inhibits anoikis [27]. The proliferative capacity of anoikis-resistant cells was also attenuated by *PIK3CA* silencing. While Adh1-EC cells demonstrated accelerated growth compared with EC cells, this phenotype was reversed in the *PIK3CA*-silenced anoikis-resistant cells. Furthermore, the clonogenic potential of Adh1-EC and EJ-*ras* EC cells remained high after loss of adhesion, whereas miR-*PIK3CA*-Adh1-EC cells exhibited a significantly reduced ability to form colonies, further confirming the reacquisition of anoikis sensitivity. According to Paoli et al. [28], non-adherent or migrating cancer cells may adopt alternative strategies to compensate for the loss of integrin signaling and overcome anoikis. These findings reinforce the dependence of anoikis-resistant cells on *PIK3CA* signaling for long-term survival following loss of adhesion.

Enhanced adhesion of anoikis-resistant endothelial cells to FN following *PIK3CA* silencing was observed, suggesting a partial recovery of normal adhesive properties. This was accompanied by a significant reduction in their invasive potential compared with the parental Adh1-EC cell line. Tumor invasion is a complex process that relies on cell adhesion, migration, and proteolytic remodeling of the ECM. This process requires dynamic interactions between invasive tumor cells and the ECM [29]. Several studies have reported that the PI3K/AKT signaling pathway promotes cellular invasion in multiple cancers [30, 31]. As the adhesion assay reflects functional adhesion rather than ECM protein expression, the unchanged adhesion to LN and Col IV following *PIK3CA* silencing may result from PI3K-independent or compensatory adhesion mechanisms. Indeed, previous studies have demonstrated context-dependent effects of PI3K suppression on Col IV adhesion [32, 33], whereas LN adhesion may be maintained by laminin-binding integrins [34].

The overexpression of caspase−3, together with increased activities of caspase−3/7 and caspase−8, was accompanied by a higher apoptotic rate in *PIK3CA*-silenced cells. AKT, activated by PI3K, promotes cell survival by phosphorylating and inactivating key apoptotic proteins, such as Bad and caspase−9, as well as by enhancing the transcription of anti-apoptotic Bcl-2 family proteins [35]. Rana and colleagues [36] demonstrated that inhibition of the PI3K/AKT pathway is associated with activation of the mitochondrial apoptotic pathway, characterized by a significant increase in the expression of pro-apoptotic Bcl-2 family members (Bad and Bax), a decrease in the anti-apoptotic protein Bcl-2, and upregulation of caspase−3 and − 9 levels [36]. In line with these findings, anoikis-resistant cells subjected to *PIK3CA* silencing showed downregulation of *BCL2* and upregulation of the pro-apoptotic genes *BAD* and *BAX*. This pattern of regulation indicates reactivation of apoptotic capacity, with restoration of anoikis sensitivity. Therefore, the reduced resazurin signal observed in *PIK3CA*-silenced cells following adhesion blockade most likely reflects increased apoptotic cell death and impaired proliferative capacity, although a contribution of reduced metabolic activity cannot be completely excluded.

Despite the novel insights provided by this study, some limitations should be acknowledged. The findings were obtained using a single in vitro anoikis-resistant endothelial cell model and should therefore be interpreted within this experimental context. Moreover, although *PIK3CA* silencing significantly affected multiple malignant characteristics, the downstream molecular mechanisms linking *PIK3CA* to ECM remodeling, cytoskeletal organization, and apoptosis remain to be fully characterized. Future studies should investigate whether these findings are conserved across different tumor and endothelial cell models. In addition, further investigation of the signaling pathways connecting *PIK3CA* to ECM remodeling and integrin-mediated signaling may provide new insights into the molecular basis of anoikis resistance and identify potential molecular targets for limiting tumor progression.

## Conclusion

Taken together, our results demonstrate that *PIK3CA* is a central regulator of multiple aspects of anoikis resistance in endothelial cells, including cell survival, clonogenic potential, proliferation, adhesion, and invasion. Silencing *PIK3CA* reprogrammed these cells toward a less aggressive phenotype, providing new insights into the molecular mechanisms underlying anoikis resistance and highlighting *PIK3CA* as a promising molecular target for future studies investigating strategies to overcome anoikis resistance and limit tumor progression.

## Data Availability

All data generated or analysed during this study are included in this published article.
